# *Nitrospina*-like Bacteria Are Dominant Potential Mercury Methylators in Both the Oyashio and Kuroshio Regions of the Western North Pacific

**DOI:** 10.1128/Spectrum.00833-21

**Published:** 2021-09-08

**Authors:** Yuya Tada, Kohji Marumoto, Akinori Takeuchi

**Affiliations:** a Department of Environment and Public Health, National Institute for Minamata Disease, Kumamoto, Japan; b Health and Environmental Risk Division, National Institute for Environmental Studies, Ibaraki, Japan; University of Minnesota

**Keywords:** mercury, methylmercury, marine bacteria, *hgcAB* genes, 16S rRNA gene, functional module

## Abstract

Highly neurotoxic methylmercury (MeHg) accumulates in marine organisms, thereby negatively affecting human and environmental health. Recent studies have revealed that oceanic prokaryotes harboring the *hgcAB* gene pair are involved in Hg methylation. Presently, little is known about the distribution and phylogeny of these genes in distinct oceanic regions of the western North Pacific. In this study, we used metagenomics to survey the distribution of *hgcAB* genes in the seawater columns of the subarctic Oyashio region and the subtropical Kuroshio region. The *hgcAB* genes were detected in the MeHg-rich offshore mesopelagic layers of both the Oyashio region, which is a highly productive area in the western North Pacific, and the Kuroshio region, which has low productivity. Comparative analysis revealed that *hgcAB* genes belonging to the *Nitrospina*-like lineage were dominant in the MeHg-rich mesopelagic layers of both regions. These results indicate that *Nitrospina*-like bacteria are the dominant Hg methylators in the mesopelagic layers throughout the western North Pacific.

**IMPORTANCE** MeHg is highly neurotoxic and accumulates in marine organisms. Thus, understanding MeHg production in seawater is critical for environmental and human health. Recent studies have shown that microorganisms harboring mercury-methylating genes (*hgcA* and *hgcB*) are involved in MeHg production in several marine environments. Knowing the distribution and phylogeny of *hgcAB* genes in seawater columns can facilitate assessment of microbial MeHg production in the ocean. We report that *hgcAB* genes affiliated with the microaerophilic *Nitrospina* lineage were detected in the MeHg-rich mesopelagic layers of two hydrologically distinct oceanic regions of the western North Pacific. This finding facilitates understanding of the microbial Hg methylation and accumulation in seawater columns of the western North Pacific.

## INTRODUCTION

Mercury is a toxic metal that is found at picomolar to nanomolar levels in the ocean. During the past century, the Hg concentration in seawater has increased due to anthropogenic emissions (1). The principal forms of Hg in seawater are Hg(0), Hg(II), and methylmercury (MeHg) (including monomethyl-Hg and dimethyl-Hg), as well as their complexes. The highly neurotoxic MeHg has been found to accumulate in marine organisms, such as plankton, fish, shells, and mammals, resulting in potentially negative impacts on human and environmental health. Thus, the vertical and horizontal distribution of Hg and MeHg in marine environments is critical for understanding MeHg accumulation in marine organisms. Ocean-scale surveys of Hg distribution have highlighted the abundance of MeHg in the mesopelagic layers (2–5). In addition, incubation studies have shown the possibility of MeHg production in seawater columns (6, 7).

Several prokaryotic lineages participate in Hg methylation in natural environments. Comparative genomic studies have revealed two key genes involved in Hg methylation, *hgcA* and *hgcB*, which encode the corrinoid [Fe-S] methyltransferase and [Fe-S]-containing ferredoxin, respectively (8). These genes are present in the genomes of various prokaryotic lineages affiliated with Deltaproteobacteria, *Bacteroidetes*, *Firmicutes*, *Chloroflexi*, *Chrysiogenetes*, *Nitrospirae*, *Nitrospinae*, *Elusimicrobia*, *Thermotogae*, *Spirochaetes*, *Aminicenantes* (candidate phylum OP8), *Atribacteria* (candidate phylum OP9), and *Euryarchaeota* (9–16). Moreover, *hgcA* and *hgcB* genes or their paralogs have been detected in several marine environments (4, 5, 10, 11, 17–19). Nevertheless, little is known about the link between MeHg distribution, the abundance of Hg-methylating genes, the phylogeny of the corresponding microorganisms in seawater columns, and the impact of diverse oceanic water bodies or currents.

Two large oceanic currents, Oyashio and Kuroshio, characterize the western North Pacific (WNP). The Oyashio region, which has a relatively low sea surface temperature and nutrient-rich waters, is highly productive (20) and important for commercial fishing of the Japanese sardine (*Sardinops melanostictus*), Japanese anchovy (*Engraulis japonicus*), and Pacific saury (*Cololabiss saira*) (21). In contrast, the Kuroshio region, which has a relatively high sea surface temperature and nutrient-poor oligotrophic waters, provides the spawning grounds for the aforementioned fish species. Thus, monitoring Hg and MeHg concentrations in seawater environments and understanding MeHg production processes such as microbial mercury methylation can benefit environmental preservation and human health. Our previous survey in the East China Sea in the Kuroshio region showed that *hgcAB* genes affiliated with the microaerophilic bacteria *Nitrospina* were abundant in the mesopelagic layers with relatively high MeHg concentrations and could contribute to MeHg accumulation (5). In comparison, there is no information about which prokaryotic lineages could be involved in MeHg production in the Oyashio region.

The purpose of this study was to examine the distribution and phylogeny of *hgcAB* genes, as well as total Hg (THg) and MeHg concentrations in the seawater column, from shore to offshore in the Oyashio region. In addition, 16S rRNA gene deep-sequencing and functional module analyses were performed to evaluate prokaryotic community structure and function. Finally, the distributions of *hgcAB* genes in the Oyashio and Kuroshio regions were compared.

## RESULTS

### Environmental characteristics.

**FIG 1:**
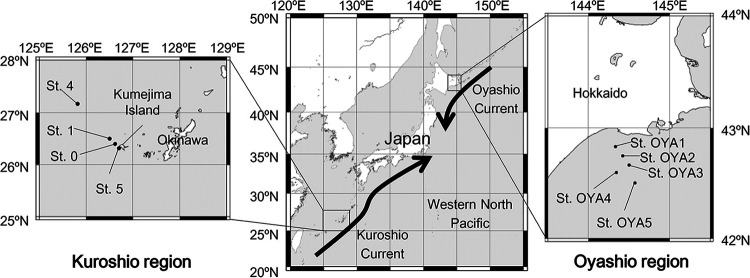
Generalized maps of the sampling sites (St.) in the Oyashio and Kuroshio regions. The sampling sites in the Kuroshio area were described by Tada et al. (5).

We collected seawater from five sites (sites OYA1 to OYA5) in the Oyashio region (Fig. 1 and Table 1). The sampling points in the Kuroshio region (5) are also shown in Fig. 1. The samples collected from the Kuroshio region were described in our previous report (5). The vertical profiles of environmental factors revealed that seawater temperature, dissolved oxygen levels, chlorophyll *a* (Chl. *a*) levels, particulate organic carbon levels, and particulate nitrogen levels decreased with depth (see Fig. S1 in the supplemental material). In contrast, the concentrations of macronutrients, including nitrate (NO_3_), phosphate (PO_4_), and silicic acid [Si(OH)_4_] increased with depth, whereas nitrite (NO_2_) levels followed a less linear pattern. The maximum number of prokaryotic cells was observed in the subsurface chlorophyll maximum (SCM) layers at sites OYA2 and OYA3 (see Table S1). Water quality profiles highlighted distinct seawater characteristics in the Oyashio and Kuroshio regions, such as low surface seawater temperature in the Oyashio region (9.7°C to 14.2°C), compared to the Kuroshio region (22.6°C to 23.2°C) (see Fig. S1a). Abundant nutrients and Chl. *a* in the Oyashio region (see Fig. S1e to h) indicated elevated biological productivity, compared to the Kuroshio region.

**TABLE 1:** Dates and locations of sampling in the Oyashio region

Site	Sampling date	Sampling location		Maximum depth (m)
		Latitude	Longitude	
OYA1	4 June 2018	42°49′N	144°19′E	277
OYA2	8 June 2018	42°45′N	144°25′E	221
OYA3	5 June 2018	42°39′N	144°30′E	540
OYA4	7 June 2018	42°35′N	144°20′E	1,070
OYA5	6 June 2018	42°30′N	144°34′E	1,750

### Vertical profiles of THg and MeHg.

**FIG 2:**
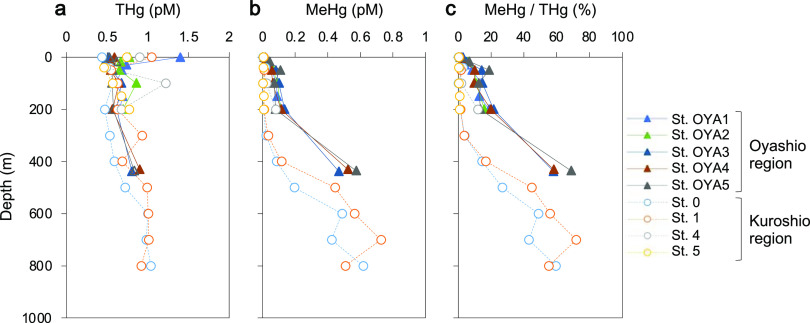
Vertical distribution of THg (a) and MeHg (b) levels and the MeHg/THg ratio (c) in the Oyashio and Kuroshio regions. Data for the Kuroshio region are from the report by Tada et al. (5).

THg levels in seawater samples ranged from 0.47 pM to 1.4 pM (Fig. 2). These values were not significantly different from those in the Kuroshio region (Student's *t* test, *P = *0.215; *n* = 29 in both regions). The concentration of MeHg and, consequently, the MeHg/THg ratio were higher in the mesopelagic layers at offshore stations. Specifically, MeHg concentrations reached 0.47 pM, 0.52 pM, and 0.57 pM (58%, 58%, and 69% of THg) at sites OYA3, OYA4, and OYA5, respectively. These findings are in line with those from previous studies showing an increase in MeHg levels in the mesopelagic layers of oceanic environments (2, 3, 5, 6, 22, 23). However, it should be noted that the maximum depth in the Oyashio region (∼430 m) was less than that in the Kuroshio region (∼800 m).

Spearman’s rank correlation analysis showed that the relationships between MeHg concentrations (and MeHg/THg ratios) and environmental factors differed from those between THg and such parameters (Table 2). THg concentrations correlated negatively with seawater temperature (rho = −0.56, *P < *0.005) and salinity (rho = −0.42, *P < *0.05). MeHg concentrations exhibited significant positive correlations with macronutrients, such as nitrate (rho = 0.91, *P < *0.001), phosphate (rho = 0.87, *P < *0.001), and silicate (rho = 0.90, *P < *0.001), as well as with apparent oxygen utilization (AOU) (rho = 0.89, *P < *0.001).

**TABLE 2:** Spearman's rank correlation analysis of concentrations of each Hg species and environmental factors in the Oyashio region

Factor^*a*^	Rho (*n* = 29)		
	THg level	MeHg level	MeHg/THg ratio
THg level	NA	NA	NA
MeHg level	0.36	NA	NA
MeHg/THg ratio	0.21	0.98^*b*^	NA
Temperature	−0.56^*b*^	−0.62^*b*^	−0.51^*b*^
Salinity	−0.43^*c*^	0.32	0.45^*c*^
Dissolved oxygen	−0.07	−0.82^*b*^	−0.86^*b*^
Chl. *a* level	−0.109	−0.69^*b*^	−0.71^*b*^
Nitrate (NO_2_) level	−0.218	0.29	0.32
Nitrite (NO_3_) level	0.40	0.91^*b*^	0.87^*b*^
Phosphate (PO_4_) level	0.57	0.87^*b*^	0.80^*b*^
Silicate (Si) level	0.49	0.90^*b*^	0.85^*b*^
POC (*n* = 17)^*d*^	−0.10	−0.40	−0.38
PN (*n* = 17)^*d*^	−0.14	−0.49^*c*^	−0.46^*c*^
PA	−0.272	−0.65^*b*^	−0.67^*b*^
AOU	0.31	0.89^*b*^	0.87^*b*^

a POC, particulate organic carbon; PN, particulate nitrogen; PA, prokaryotic abundance; NA, not applicable.

b *P* < 0.01.

c *P* < 0.05.

d The number used for statistical analysis.

### Relative abundance and distribution of *recA* and *hgcAB* genes in the seawater column.

The abundance of *hgcAB* was estimated based on metagenomic sequences and was normalized to that of the *recA* gene (24), an essential single-copy gene in bacteria. The abundance of *recA* ranged from 0.04% to 0.06% of total predicted genes (Table 3). The *hgcA* sequences were detected only in the mesopelagic layers (∼450 m) at the offshore stations (OYA3, OYA4, and OYA5), whereby they accounted for 0% to 0.46% of the abundance of *recA* genes, with the greatest proportions corresponding to offshore stations. The most abundant *hgcB* sequence was observed at a depth of 436 m at the offshore station OYA5, accounting for 0.70% of the abundance of the *recA* gene.

**TABLE 3:** Abundance of total predicted genes, *recA*, and *hgcAB* in the metagenomic contigs

Parameter	Data for site^*a*^:																						
	OYA1				OYA2				OYA3					OYA4					OYA5				
	0 m	30 m (SCM)	100 m	200 m	0 m	18 m (SCM)	100 m	200 m	0 m	20 m (SCM)	100 m	200 m	438 m	0 m	15 m (SCM)	100 m	200 m	431 m	0 m	16 m (SCM)	100 m	200 m	436 m
No. of predicted genes (>30 amino acids)	1,116,047	1,150,203	1,763,051	ND	823,907	1,242,923	1,306,632	1,622,910	1,361,227	1,813,231	1,947,062	1,980,915	1,430,724	ND	ND	2,098,548	1,867,205	1,468,159	ND	ND	1,854,873	1,740,418	1,659,884
No. of *recA* sequences	465	501	957	ND	384	595	786	1,007	697	738	991	1,183	732	ND	ND	1,109	1,037	804	ND	ND	1,081	1,007	861
No. of *hgcA* sequences	0	0	0	ND	0	0	0	0	0	0	0	0	1	ND	ND	0	0	2	ND	ND	0	0	4
No. of *hgcB* sequences	0	0	4	ND	0	0	4	4	0	0	6	6	4	ND	ND	6	5	4	ND	ND	7	3	6
Relative *recA* abundance (% of total predicted genes)	0.042	0.044	0.054	ND	0.047	0.048	0.060	0.062	0.051	0.041	0.051	0.060	0.051	ND	ND	0.053	0.056	0.055	ND	ND	0.058	0.058	0.052
Relative *hgcA* abundance (% of *recA* abundance)	0	0	0	ND	0	0	0	0	0	0	0	0	0.14	ND	ND	0	0	0.25	ND	ND	0	0	0.46
Relative *hgcB* abundance (% of *recA* abundance)	0	0	0.42	ND	0	0	0.51	0.40	0	0	0.61	0.51	0.55	ND	ND	0.54	0.48	0.50	ND	ND	0.65	0.30	0.70

a ND, not determined.

### Phylogeny of *hgcAB* genes.

**FIG 3:**
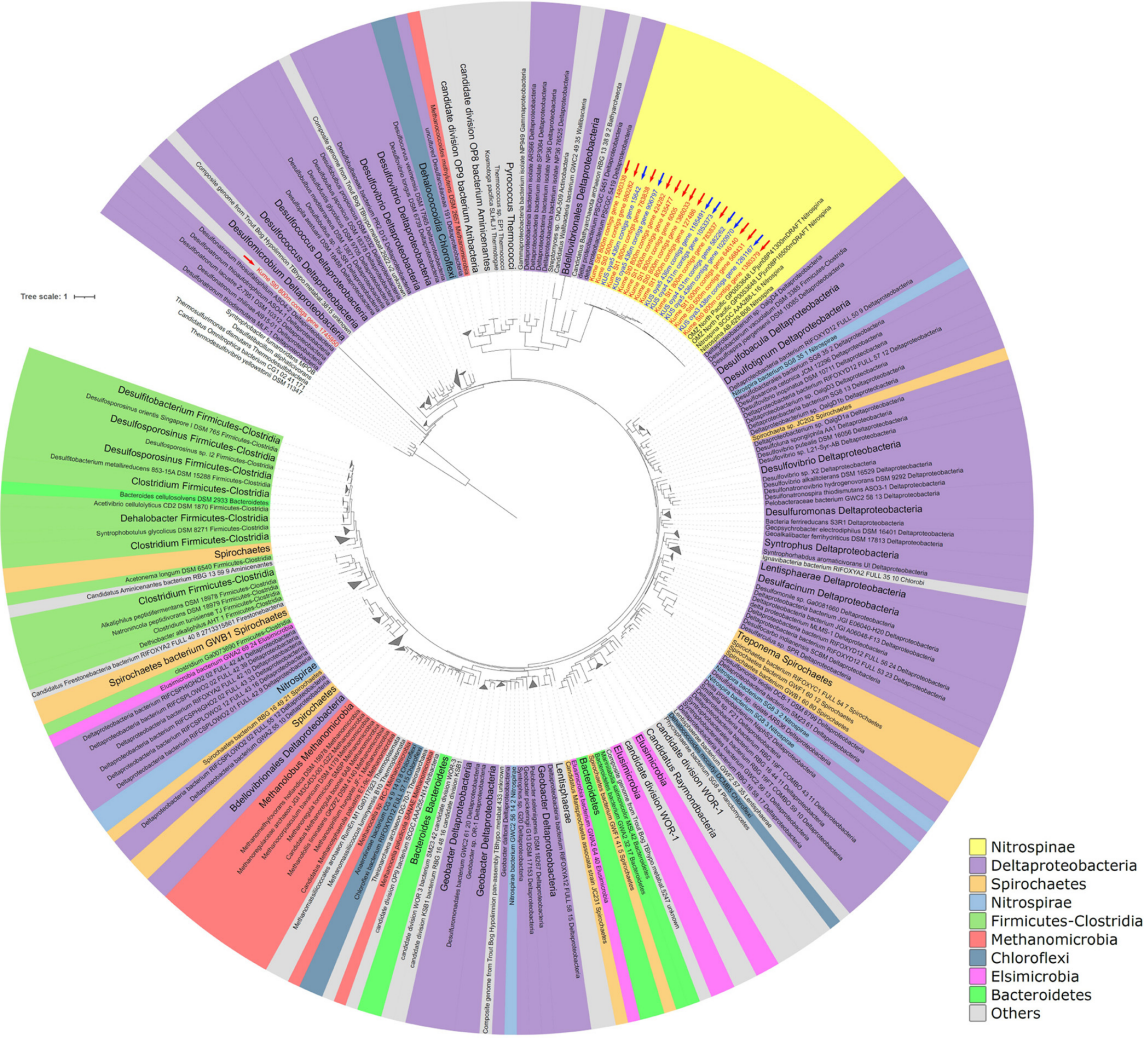
Maximum likelihood tree of *hgcA* sequences identified in the Oyashio and Kuroshio regions (blue and red arrows, respectively). Sequences identified in this study were compared to *hgcA* homologs described previously (16). The tree is rooted by *hgcA* paralogs in nonmethylators. The scale bar represents substitutions per site. Data for the Kuroshio region are from the report by Tada et al. (5).

HMMER analysis detected 7 *hgcA* sequences (2 complete sequences and 5 partial sequences) with a conserved cysteine C93 motif (8, 11, 25) in the mesopelagic layers at offshore stations (see Table S3). Compared to *hgcA* sequences previously obtained from methylator and metagenomic data (16), all *hgcA*-like sequences detected in the Oyashio region were closely related to those in *Nitrospina*, a species affiliated with *Nitrospinae* (Fig. 3). Interestingly, these *Nitrospina*-like *hgcA* sequences were closely related to those detected in the Kuroshio region (5). Similarly, numerous *hgcB* sequences were also affiliated with *Nitrospina*-like lineages and were akin to those detected in the Kuroshio region (see Fig. S2).

### Depth profiles of the prokaryotic community.

To evaluate prokaryotic community composition in greater detail, we performed 16S rRNA gene deep-sequencing analysis with primers targeting the V4 region (26, 27). The resulting depth profiles of phylogenetic lineages, such as phyla and classes, included established or predicted Hg methylators (see Fig. S3). The relative abundances of *Euryarchaeota*, Deltaproteobacteria (this phylum was recently reclassified into four phyla, namely, *Desulfobacterota*, *Myxococcota*, *Bdellovibrionota*, and SAR324 [28]), and *Chloroflexi* increased below the subsurface layers and accounted for 5.8% to 6.7%, 14.6% to 19.8%, and 1.4% to 1.6%, respectively, of the total 16S rRNA gene sequences in the mesopelagic layers (∼430 m in depth). In contrast, no specific patterns were observed for the *Nitrospinae* and *Firmicutes* lineages. Based on rarefaction curves of operational taxonomic units (OTUs) (>97% sequence similarity) in the Oyashio region, the greatest abundance of other phylogenetic lineages was detected at a depth of ∼430 m at sites OYA3 (1,335 OTUs) and OYA4 (1,356 OTUs) (see Fig. S4a).

### Distribution of functional modules.

**FIG 4:**
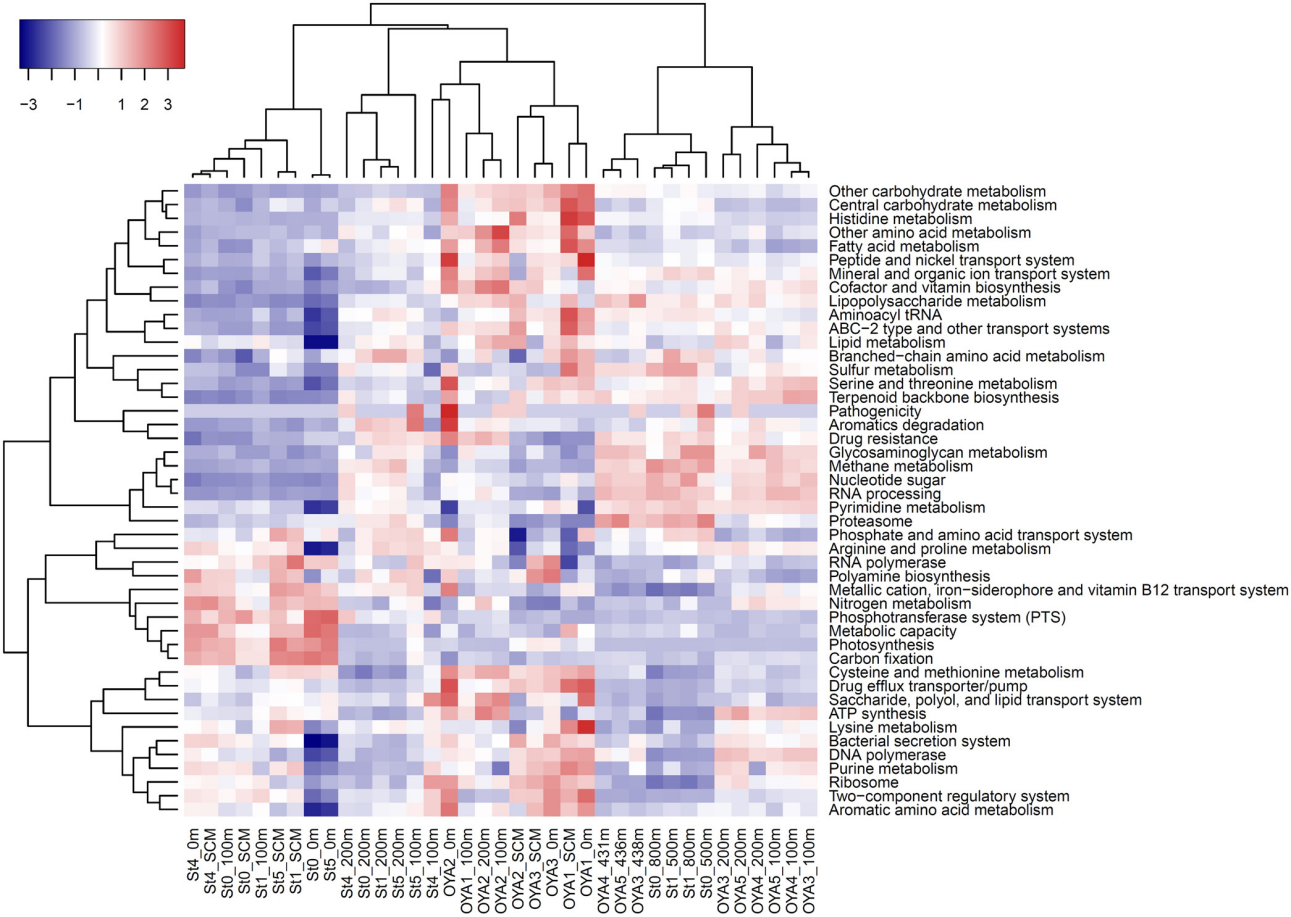
Heatmap diagram indicating relative abundance of metabolic modules in the Oyashio and Kuroshio regions. Euclidean distance was used for distance calculations among treatments (both sampling sites and functional modules) after data normalization. Data for the Kuroshio region are from the report by Tada et al. (5).

Information about functional genes and modules is important for evaluating the ecological function and metabolism of prokaryotes, including mercury methylators. We estimated the abundance of prokaryotic functional modules in the metagenomic sequences using a physiological potential evaluator (Genomaple [https://hub.docker.com/r/genomaple/genomaple]) (29), with a gene and module database (version dated 13 July 2018) defined by the Kyoto Encyclopedia of Genes and Genomes (KEGG). This software automatically mapped genes in metagenomic sequences to 796 functional modules in the KEGG database and calculated the module completion ratio in each functional module. The composition of prominent functional modules differed between surface and mesopelagic layers (below 431 m in the Oyashio region and below 500 m in the Kuroshio region) (Fig. 4). In the surface layers, the dominant modules were involved in photosynthesis, as well as fatty acid, cysteine, and methionine metabolism. In contrast, nucleotide sugar, RNA processing, methane metabolism, glycosaminoglycan metabolism, and proteasome modules seemed to be abundant in deeper waters.

## DISCUSSION

### THg and MeHg distributions in the Oyashio region.

Assessment of THg and MeHg levels in the Oyashio region revealed strong positive correlations between the MeHg concentrations (and MeHg/THg ratios) and nutrient (e.g., phosphate, silicate, and nitrate) amounts or AOU (Table 2). This finding suggested a strong link between MeHg production and organic matter remineralization by microorganisms (22). Previous surveys in the Mediterranean Sea and Pacific Ocean revealed similar trends between MeHg and macronutrients (especially phosphate) (2, 30). These data indicate that marine microorganisms are critical producers of MeHg in seawater columns.

In both the Oyashio and Kuroshio regions, significant positive correlations between MeHg concentrations and AOU values were observed, suggesting that Hg methylation correlated with microbial activity (remineralization and respiration). The MeHg concentration was higher in the Oyashio region (median, 0.070 pM) than in the Kuroshio region (median, 0.015 pM). In addition, *hgcAB* genes were detected in seawater with relatively high MeHg concentrations. Taken together, the contributions of marine microorganisms to MeHg production in the seawater column could differ between oceanic regions with distinct seawater characteristics.

### Distribution and phylogeny of *hgcAB* genes.

Phylogenetic analyses identified *hgcAB* genes and *Nitrospina-*like genes in the MeHg-rich mesopelagic layers of both regions. These data suggest that aerobic *Nitrospina* lineages could be involved in Hg methylation in the mesopelagic layers of the WNP. Some *Nitrospina* lineages are known nitrite oxidizers under aerobic and anaerobic conditions, such as mesopelagic layers in the ocean (31, 32). Previous metagenomic analyses detected *Nitrospina*-like *hgcA* genes in several marine environments, such as Antarctic Sea ice, the equator, and the East China Sea (4, 5, 11, 18). Phylogenetic analysis also identified *Nitrospina*-like *hgcB* sequences in both the Kuroshio and Oyashio regions (see Fig. S2 in the supplemental material). These data suggest that *Nitrospina* lineages participate extensively in MeHg production throughout the ocean.

To date, Hg methylation by *Nitrospina* lineages has not been confirmed, as no *Nitrospina* culture has been isolated. In addition, there is no evidence of a relationship between *Nitrospina* lineages and Hg methylation in natural environments, such as sediments, freshwater, and seawater. Thus, a combination of methods, including isolation, incubation with isotopes, and metagenomic and metatranscriptomic analyses, will be necessary to evaluate MeHg production by *Nitrospina* lineages.

Previous metagenomic studies on seawater samples identified *hgcAB* genes in sulfate-reducing bacteria, iron-reducing bacteria, and methanogens affiliated with Deltaproteobacteria, *Spirochaetes*, and *Euryarchaeota* lineages in the Baltic Sea (17); Deltaproteobacteria, *Firmicutes*, and *Chloroflexi* in the oceans (except for the western part of the Pacific Ocean) (18); and *Calditrichaeota*, SAR324, and *Marinimicrobia* in the Saanich Inlet (19). However, *hgcAB* sequences affiliated with these anaerobic microorganisms were not detected in our metagenomic data, suggesting limited contributions of these lineages to MeHg production in the seawater column of the WNP. This discrepancy could be explained by the different water characteristics in the Oyashio and Kuroshio regions, compared to the Baltic Sea (a brackish inland sea) and the Saanich Inlet (a seasonally anoxic fjord). Distinct seawater properties can translate into different microbial communities and types of Hg methylation. Another explanation is the relatively small volume of data for our query sequences. In general, detection efficiency depends strongly on the abundance and quality of raw query sequences. For example, the Baltic Sea metagenomic data contain over 22 million contigs (average of 69.5 million paired-end reads per sample) (33), whereas our metagenome samples contained 0.74 to 1.79 million contigs (see Table S2). Thus, more numerous and high-quality sequences (at least 10 to 20 GB) are necessary to evaluate the abundance and diversity of *hgcAB* genes (especially *hgcA*) in the open ocean. For now, our metagenomic survey could estimate the dominant Hg methylators in the Oyashio and Kuroshio regions.

### Prokaryotic community composition.

Deep-sequencing analysis showed that the greatest abundance of the *Nitrospinae* 16S rRNA gene did not coincide with the mesopelagic layers rich in MeHg and *hgcAB* genes. These inconsistencies could be due to the resolution of phylogenetic analyses based on partial 16S rRNA gene deep sequencing. Community analysis with a partial 16S rRNA gene could detect *Nitrospina* species harboring or not harboring (e.g., Nitrospina gracilis) the *hgcAB* genes. These data suggest that both 16S rRNA gene analysis and metagenomic studies are indispensable for evaluating microbial Hg methylation in the ocean.

Furthermore, 16S rRNA gene analyses revealed that Deltaproteobacteria, *Firmicutes*, and *Euryarchaeota*, including several known Hg methylators, were dominant in the mesopelagic layers with high MeHg concentrations. However, *hgcAB* genes affiliated with these lineages were not detected at the same depth, suggesting the limited contribution of these phylogenetic lineages to microbial MeHg production in the mesopelagic layers of the Oyashio region. Detailed analysis of 16S rRNA sequences revealed that approximately 20% of Deltaproteobacteria strains were affiliated with the uncultured Sva0853 lineage (see Fig. S5b), which is abundant in the free-living fraction of the mesopelagic layer (34). To date, however, the *hgcAB* genes and Hg methylation ability of these lineages have not been confirmed. Similarly, no Hg methylators belonging to the *Firmicutes* and *Euryarchaeota* were observed in the 16S rRNA gene sequences.

### Distribution of functional modules.

Functional module analyses using Genomaple showed that the modules for methane and sulfur metabolism were relatively abundant in the mesopelagic layers of the Oyashio region. However, *hgcAB*-like sequences belonging to methanogens and sulfate-reducing bacteria were not detected in these layers. The same trend was observed previously in the Kuroshio region (5), indicating the negligible contribution of these members to MeHg production in the mesopelagic layers of both regions. Taken together, these data point to potentially similar microbial Hg methylation processes (presumably by the *Nitrospina* lineage) in two major currents of the WNP.

### Conclusion.

Comparison of metagenomic data obtained from the hydrologically distinct Oyashio and Kuroshio regions revealed that *hgcAB* genes were abundant in the MeHg-rich mesopelagic layers of both regions. Phylogenetic analyses showed that the *hgcAB* genes detected in both regions were closely related to *Nitrospina*-like genes. These data suggest that *Nitrospina*, a microaerophilic bacterium, can be a potential Hg methylator and is involved in MeHg production in the mesopelagic layers of the WNP.

## MATERIALS AND METHODS

### Seawater sampling and hydrological conditions.

We collected seawater from five sampling sites in the Oyashio region. Detailed information about sampling locations, sampling dates, and maximum depths are presented in Table 1. Seawater samples were collected using acid-washed (1 M HNO_3_) clean Niskin samplers with Teflon-coated interior walls. The detailed procedures for sampling and determination of THg and MeHg levels were described by Tada et al. (5). Briefly, approximately 1 liter of seawater was collected in 1-liter acid-washed Teflon bottles; 800 ml was transferred to acid-washed Teflon bottles containing ultrapure H_2_SO_4_ (final concentration, 0.5 M) for MeHg analysis, whereas 200 ml was transferred to 250-ml acid-washed Teflon bottles and mixed with 0.5% concentrated ultrapure HCl plus 0.2 M BrCl (final concentration) for THg analysis. The treated samples were stored at 4°C in the dark until further analysis.

For metagenomic analysis, 10 liters of seawater was collected in a sterile plastic container and stored at ∼10°C in the dark until filtration, which occurred within 8 h after sample collection. Samples were filtered through a 0.22-μm-pore-size Sterivex-GP cartridge filter (Millipore, Billerica, MA, USA) to collect prokaryotic cells. The cartridge filters were stored at −80°C until further treatment. DNA extraction procedures are described in the supplemental material.

Physicochemical parameters of seawater, including temperature, salinity, Chl. *a* levels, and dissolved oxygen concentrations were measured using a CTD (conductivity, temperature, and depth) system (RINKO-Profiler; JFE Advantec Co., Ltd., Nishinomiya, Japan). The depth of SCM layers was determined based on the Chl. *a* profile. Samples were collected to evaluate the concentrations of macronutrients, including NO_3_ plus NO_2_, PO_4_, and Si(OH)_4_, as well as particulate organic carbon and particulate nitrogen. Detailed procedures are described in the supplemental material. Prokaryotic cells were counted under an epifluorescence microscope after staining with SYBR Gold (35). At least 10 microscope fields were counted per sample.

### THg and MeHg analyses.

THg and MeHg levels were determined according to the protocols described by Marumoto et al. (36). Briefly, THg was measured following EPA method 1631 (37). The concentrations of THg were determined using cold vapor atomic fluorescence spectrometry and gold amalgamation (RA-FG^+^; Nippon Instruments Co., Tokyo, Japan) following the generation of Hg(0) using 1 ml of 20% (wt/vol) SnCl_2_ as a reducing agent. The precision of the THg assay was verified by performing multiple measurements of the BCR579 standard reference material (certified range of 9.5 ± 2.5 pM). We found that the determined values (9.3 ± 0.3 pM [*n *= 6]) did not vary within this range. The method’s detection limit was determined using blank solutions with ultrapure water and reagents and was calculated as 0.090 pM (*n *= 4), which corresponded to 3 times the standard deviation of the blank. Multiple measurements of the blank showed that the THg concentration was 0.069 ± 0.030 pM (*n *= 4). The blank value of the reagent (BrCl solution) was 0.017 pM and was calculated with gradual changes in the reagent volume. The THg concentration in seawater samples was calculated by subtracting the reagent blank value.

The procedure used to determine MeHg levels was based on solvent extraction with dithizone-toluene and Na_2_S (38). The MeHg concentrations in Na_2_S solutions were determined via ethylation using NaB(C_2_H_5_)_4_, preconcentration onto a Tenax trap, thermal desorption, and gas chromatography with atomic fluorescence detection, as described by Logar et al. (39). The method’s detection limit was calculated analogously as for THg and was estimated as 0.006 pM (*n *= 8) with respect to the blank (ultrapure water). The value for the blank was 0.004 ± 0.002 pM (*n *= 3). MeHg recovery was 99% ± 3% (*n *= 7), based on a spike of a known concentration of MeHg obtained from alkaline dissolution of DORM-2 (certified range, 4.47 ± 0.32 mg/kg dry weight), a standard reference material for MeHg in dogfish. At least one DORM-2 solution was measured for every five MeHg sample measurements.

### 16S rRNA gene deep sequencing.

Prokaryotic V4 small subunit rRNA gene fragments were amplified from the extracted DNA using the primer set 515F (5′-GTG CCA GCM GCC GCG GTA A-3′) and 806RB (5′-GGA CTA CNV GGG TWT CTA AT-3′) (26, 27) combined with an Illumina (San Diego, CA, USA) adapter. The PCR amplicons were sequenced over two 300-bp paired-end Illumina MiSeq runs.

### Metagenome sequencing.

Metagenomic DNA (approximately 250 ng) was barcoded by sample and used for library preparation. Paired-end libraries (insert size of ∼350 bp) were generated using the NEBNext Ultra DNA library preparation kit according to the manufacturer’s instructions (New England Biolabs, Ipswich, MA, USA). Sequencing was performed by Novogene Bioinformatics Technology (Beijing, China) using Illumina HiSeq 2500 paired-end (2 × 250-bp) sequencing. DNA sequencing of several samples (collected from a 200-m depth at site OYA1 and from a 0-m depth and SCM at sites OYA4 and OYA5) failed, presumably because of insufficient quality of the extracted DNA (see Table S2 in the supplemental material).

### Sequence data analyses.

After Illumina adaptor sequences were removed using Cutadapt (40), metagenomic contigs were constructed using MEGAHIT (41) with default parameters (k-min = 21, k-max = 141, k-step = 12, t = 20). To predict the genes in the metagenomic sequences, we used MetaGeneAnnotator (42), after which putative open reading frame reads were extracted and translated into amino acid sequences using a bacterial translation table (transl_table = 11). To detect *recA* and *hgcAB* sequences in our metagenomes, a hidden Markov model (HMM) profile was constructed using HMMER v3.2.1 (43) with an E value cutoff of 10^−5^. The *recA* HMM reference was constructed using PF00154 as the representative protein sequence. To develop the HMM profile for the *hgcAB* gene pair (16), the two genes were aligned using MEGA-X software (44). We slightly modified the *hgcB* HMM profile with the addition of *Nitrospina*-like *hgcB* genes (11). To recheck the specificity of HMMs for these sequences, a local search with the hmmsearch tool (HMMER v3.2.1) was performed with the reference sequences. Finally, *hgcA*-like sequences without the conserved C93 cysteine (8, 11, 25) were trimmed, whereas *hgcB* sequences without two strictly conserved CX_2_CX_2_CX_3_C motifs were removed (11, 25).

Maximum likelihood trees of *hgcAB* were constructed using FastTree (45) with 1,000 ultrafast bootstrap replicates after sequence alignment with MEGA-X. The trees were visualized using iTOL (https://itol.embl.de) (46).

For the Genomaple analysis, we used the refined paired-end assembly sequences in PEAR (47), FastXToolKit (http://hannonlab.cshl.edu/fastx_toolkit), and PRINSEQ (48). The details of the corresponding procedures were described by Tada et al. (5).

The 16S rRNA gene analyses were completed in the QIIME 2 pipeline (https://qiime2.org). Chimeras were removed using USEARCH (49) with the reference Greengenes 16S rRNA gene data set (50). After quality filtration, sequences were clustered into OTUs with a 97% similarity threshold.

### Statistical analyses.

Data were analyzed with R software v.3.4.3 (51), using Spearman’s rank correlation to investigate the relationship between *hgcAB* abundance and various environmental factors, including THg and MeHg levels. A heatmap of functional module abundance was constructed in R after standardization.

### Data availability.

Raw metagenomic sequence data were deposited in the DNA Data Bank of Japan (DDBJ) Sequence Read Archive (accession number DRA012127). Sequence data for the 16S rRNA genes (accession number DRA012128) were also archived in the same repository.
