# Supplementary material for: Nitrospina-like Bacteria Are Dominant Potential Mercury Methylators in Both the Oyashio and Kuroshio Regions of the Western North Pacific
Source: Microbiol Spectr. 2021 Sep 8;9(2):e00833-21. doi: 10.1128/Spectrum.00833-21 (PMC8557936; doi:10.1128/Spectrum.00833-21)
Supplement: SUPPLEMENTAL FILE 1 — Supplemental material. Download SPECTRUM00833-21_Supp_1_seq1.pdf, PDF file, 0.3 MB. [file spectrum00833-21_supp_1_seq1.pdf]

Supporting information

Table S1. Raw data of environmental factors

Station name	Depth (m)	Remarks	Seawater temperature [°C]	Salinity [PSU]	THg [pM]	MeHg [pM]	MeHg/THg [%]	DO [ml l^{-1}]	AOU [ml l^{-1}]	Chl. *a* [μg l^{-1}]	Nitrite [μM]	Nitrate [μM]	Phosphate [μM]	Silicate [μM]	POC [μg l^{-1}]	PN [μg l^{-1}]	Prokaryotic cell abundance [×10^5 cells ml^{-1}]
OYA1	0		8.9	29.58	1.4	0.0	1.1	8.49	-1.72	0.77	ND	ND	0.71	ND	149.7	21.5	1.00
	30	SCM	3.0	32.85	0.7	0.0	4.0	8.77	-1.16	2.17	0.01	1.03	0.46	ND	225.5	37.9	0.78
	50		2.1	33.12	0.7	0.1	8.4	6.91	0.86	0.96	0.17	16.50	1.66	28.97	No data	No data	No data
	100		3.3	33.46	0.6	0.1	14.0	6.44	1.07	0.18	0.18	19.76	1.67	31.91	52.4	3.5	0.56
	150		2.8	33.45	0.7	0.1	12.8	6.35	1.26	0.19	0.15	22.16	1.78	39.49	No data	No data	No data
	200		2.6	33.51	0.7	0.1	18.8	6.03	1.62	0.17	0.15	26.44	2.02	50.37	64.5	3.9	0.67
OYA2	0		9.7	32.57	0.8	0.0	1.2	7.98	-1.48	0.35	ND	ND	0.39	ND	183.3	29.8	0.75
	18	SCM	3.3	33.01	0.7	0.0	5.9	8.78	-1.23	10.66	0.10	9.77	1.21	12.66	244.9	41.0	1.31
	50		1.4	33.08	0.7	0.1	9.9	6.71	1.20	0.7	0.11	18.99	1.83	37.45	No data	No data	No data
	100		1.5	33.23	0.9	0.1	10.8	6.22	1.66	0.25	0.16	22.44	1.95	41.71	69.5	5.7	1.10
	200		2.9	33.44	0.7	0.1	15.9	6.10	1.50	0.4	0.16	21.77	1.81	38.68	61.0	4.6	1.14
OYA3	0		12.4	33.85	0.5	0.0	3.0	6.67	-0.59	0.75	0.00	0.04	0.17	ND	276.8	44.8	1.05
	18	SCM	10.5	33.64	0.5	0.0	5.0	7.06	-0.71	3.63	0.11	3.72	0.43	ND	156.7	24.2	1.49
	50		7.8	33.93	0.6	0.1	14.4	5.90	0.84	0.19	0.37	13.11	1.09	14.23	No data	No data	No data
	100		4.9	33.62	0.7	0.1	14.8	6.24	0.99	0.13	0.02	16.52	1.28	24.04	45.9	2.3	0.70
	200		1.5	33.37	0.6	0.1	21.6	5.82	2.06	0.16	0.11	27.20	2.11	52.19	43.1	2.1	0.75
	438		3.1	33.86	0.8	0.5	58.1	2.55	4.99	0.17	0.03	37.73	2.70	85.40	40.5	1.4	0.44
OYA4	0		14.2	33.75	0.6	0.0	1.4	6.56	-0.69	0.55	ND	ND	0.10	ND	273.8	44.8	1.72
	16	SCM	10.1	33.82	0.6	0.0	2.7	6.82	-0.43	3.54	0.13	3.11	0.31	ND	178.4	29.1	0.78
	50		8.0	33.85	0.5	0.1	10.1	6.27	0.43	0.37	0.38	10.27	1.00	10.05	No data	No data	No data
	100		5.9	33.76	0.6	0.1	9.7	5.94	1.11	0.13	0.03	13.30	1.04	18.97	41.8	1.6	0.97
	200		5.3	33.82	0.6	0.1	19.8	5.79	1.35	0.1	0.04	15.82	1.20	26.07	38.2	0.7	0.38
	431		3.4	33.91	0.9	0.5	58.2	2.33	5.16	0.14	0.03	38.42	2.77	80.60	No data	No data	0.49
OYA5	0		13.1	33.77	0.5	0.0	2.2	6.56	-0.55	0.99	0.10	2.08	0.24	ND	No data	No data	1.98
	16	SCM	10.6	33.87	0.6	0.0	6.7	6.72	-0.40	4.72	0.14	2.98	0.30	ND	No data	No data	0.87
	50		9.3	34.21	0.6	0.1	18.8	5.53	0.96	0.19	0.24	12.44	0.97	15.33	No data	No data	No data
	100		7.2	34.07	0.6	0.1	12.5	6.12	0.69	0.12	0.02	11.19	0.85	14.44	No data	No data	0.92
	200		4.9	33.95	0.6	0.1	13.5	4.78	2.42	0.12	0.02	16.53	1.27	26.32	No data	No data	0.48
	436		3.7	34.38	0.8	0.6	68.9	1.03	6.36	0.17	0.01	38.16	2.72	86.11	No data	No data	0.44

Abbreviations: SCM, Subsurface chlorophyll maximum; THg, Total Hg; MeHg, Methylated Hg; DO, Dissolved oxygen; AOU, Apparent oxygen utilization; Chl. *a*, Chlorophyll *a*; POC, Particulate organic carbon; PN, Particulate nitrogen

ND: Not detected

Table S2. Detail sequence information

Metagenomic samples	St. OYA1				St. OYA2				St. OYA3					St. OYA4					St. OYA5				
	0 m	30 m (SCM)	100 m	200 m	0 m	18 m (SCM)	100 m	200 m	0 m	20 m (SCM)	100 m	200 m	438 m	0 m	15 m (SCM)	100 m	200 m	431 m	0 m	16 m (SCM)	100 m	200 m	436 m
Quantitiy of metagenomic DNA (ng)	1058	1752	3240	2472	3240	2700	3240	3300	1686	1482	1074	996	642	834	840	816	690	402	411	585	672	972	288
Volume of sequence data (GB)	7.6	7.8	7.3	ND	5.9	7.4	5.8	7.1	6.8	7.3	8	7.5	6.9	ND	ND	8.4	6.7	6.4	ND	ND	6.8	7	7.2
No. of contigs after MEGAHIT assmbly**	983398	1027863	1615141	ND	735186	1201299	1148017	1440638	1226640	1737378	1688759	1684282	1360234	ND	ND	1793950	1591382	1236630	ND	ND	1542490	1439951	1448177
N50 of contigs (bp)	494	496	530	ND	576	519	570	537	556	484	539	541	537	ND	ND	539	548	563	ND	ND	558	569	540
Minimum length of contigs (bp)	200	200	200	ND	200	200	200	200	200	200	200	200	200	ND	ND	200	200	200	ND	ND	200	200	200
Maximum length of contigs (bp)	17584	17799	42610	ND	88410	66097	48755	63151	33817	10317	36379	26446	35148	ND	ND	50079	40242	46623	ND	ND	42441	33255	59395
Average length of contigs (bp)	506	505	535	ND	583	539	570	545	556	492	542	540	538	ND	ND	541	549	556	ND	ND	555	564	544
Mapped reads																							
Coverage of mapped read (percentage of total reads)	41.9	49.69	67.65	ND	77.76	70.51	69.62	51.72	67.09	49.53	68.88	67.58	59.62	ND	ND	68.75	64.81	66.24	ND	ND	69.36	64.29	67.77
16S rRNA amplicon sequence reads	29150	25457	24980	25643	29113	29593	23605	23548	28959	29759	37468	41249	46483	45387	39870	30145	43241	44292	38290	43627	44137	45318	23048

*Remove the low quality sequences after paired-end assembly

**Assemble condition (k-min = 21, k-max = 141, and k-step = 12)

ND: No data

Table S3. *hgcA* sequences detected in the Oyashio region

Contig name	*hgcA* sequence detected in this study (amino acid sequence)
KUS_oya3_438m_contigs_1261167_c	MINWIKDIYQTLFRFARFPCEPETVTIGNPDKSSPVLVTCNFDYTVRHLKEYLKKEALDCFLLVVNTKGT**NVWCAAAEG**VFTTDIVLSHLKVYNVGELVNHKRLILPQLSVAGVKRKELKEHGWEGIYGPVYFTDLKEFLNNGLTKNKDMQALEYGYWERFKMSLSHAVFCTLVCIIPIFLFASDWWIQGIGLVWYFAFSMQLIEHFIPFERLLYKGLALSLPILVLTLTSITDPVLKTQATLGVIALGGYIGYDAQGHSHLGQNQKSGTIFAGMFAFLALVYGGTLFL
KUS_oya4_431m_contigs_582262_c	MINWIKDIYQTLFRFARFPCEPETVTIGNPDKSSPVLVTCNFDYTVRHLKEYLKKEALDCFLLVVNTKGT**NVWCAAAEG**VFTTDIVLSHLKVYNVGELVNHKRLILPQLSVAGVKRKELKEHGWEGIYGPVYFTDLKEFLNNGLTKNKDMQALEYGYWERFKMGLSHAVFCTLVCIIPIFLFASDWWIQGIGLVWYFAFSMQLIEHFIPFERLLYKGLALSLPILVLTLTSITDPALKTQATLGVIALGGYIGYDAQGHSHLGQNQKSGTIFAGMFAFLALVYGGTLFL
KUS_oya4_431m_contigs_1053373_p	EKEQLDCFLLVVNTQGT**NVWCAAAEG**IFTTETVLAHLKVYNVNDMVDHTRLILPQLSVAGIKRKDLKEHGWEGIYGPVYFTDLKEFLKNGLTKTKEMQALEYGYWERFKMGLSHAVFCTLVCILPIFLFASDWWLQAIALVWYFAFSMQLIEHFIPFHRLLYKGLALTLPVLAIVLFSVTDSVLRIQATIGIIALGAYIGYDAQGHSHLGQNQQSGKLFARIFASLALIYGGTLLL
KUS_oya5_436m_contigs_15642_p	MIGWIKDIYQTLFRFARFPCEPETIAIGSPDKSSPILVTCNFDYTVRHLKEYLKKEALDCFLLVVNTKGT**NVWCAAAEG**VFTTDTVLSHLKVYNVGELVNHKRLILPQLSVAGVKRKE
KUS_oya5_436m_contigs_906797_p	TVLIGNPDKSSPVLVTCNFDYTVRHLKDYLEKEKLDCFLLVVNTKGT**NVWCAAAER**IFTTDTVLSHLKVYNVGELVNHHQLILPQLSVAGVKRKELKEHGWEGIYGPVYFADLKEFLNNGLTKNKDMQALEYGYWERFKMGLSHAVFCALVCIVPILFFASDY
KUS_oya5_436m_contigs_1020970_p	MINWIKDIYQTLFRFARFPCEPETVTIGNPDKSSPVLVTCNFDYTVRHLKEYLKKEALDCFLLVVNTKGT**NVWCAAAEG**VFTTDIVLSHLKVYNVGELVNHKRLILPQLSVAGVKRKELKEHGWEGIYGPVYFTDLKEFLNNGLTKNKDMQALEYGYWERFKMGLSHAVFCTLVCIIPIFLFASDWWIQGIGLVWYFAFSMQLIEHFIPFERLLYKG
KUS_oya5_436m_contigs_1185491_p	MIGWIKDIYQTLFRFARFPCEPETVSVGNPDKSSPVLVTCNFDYTVRHLKKYLEKEQLDCFLLVVNTQGT**NVWCAAAEG**IFRTETVLAHLRVYNVNDMVDHTRLILPQLSVAGIKRKDLKEHGWEGIYGPVYFTDLKEFLKNGLTKTKEMQALEYGYWERFKMGLSHAVFCTLVCILPIFLFASDWWLQAIALVWYFAFSMQLIEHFIP

Bold letters indicate the putative cap helix region
