# Supplementary material for: Nitrospina-like Bacteria Are Dominant Potential Mercury Methylators in Both the Oyashio and Kuroshio Regions of the Western North Pacific
Source: Microbiol Spectr. 2021 Sep 8;9(2):e00833-21. doi: 10.1128/Spectrum.00833-21 (PMC8557936; doi:10.1128/Spectrum.00833-21)
Supplement: SUPPLEMENTAL FILE 2 — Supplemental material. Download SPECTRUM00833-21_Supp_2_seq5.pdf, PDF file, 1.7 MB. [file spectrum00833-21_supp_2_seq5.pdf]

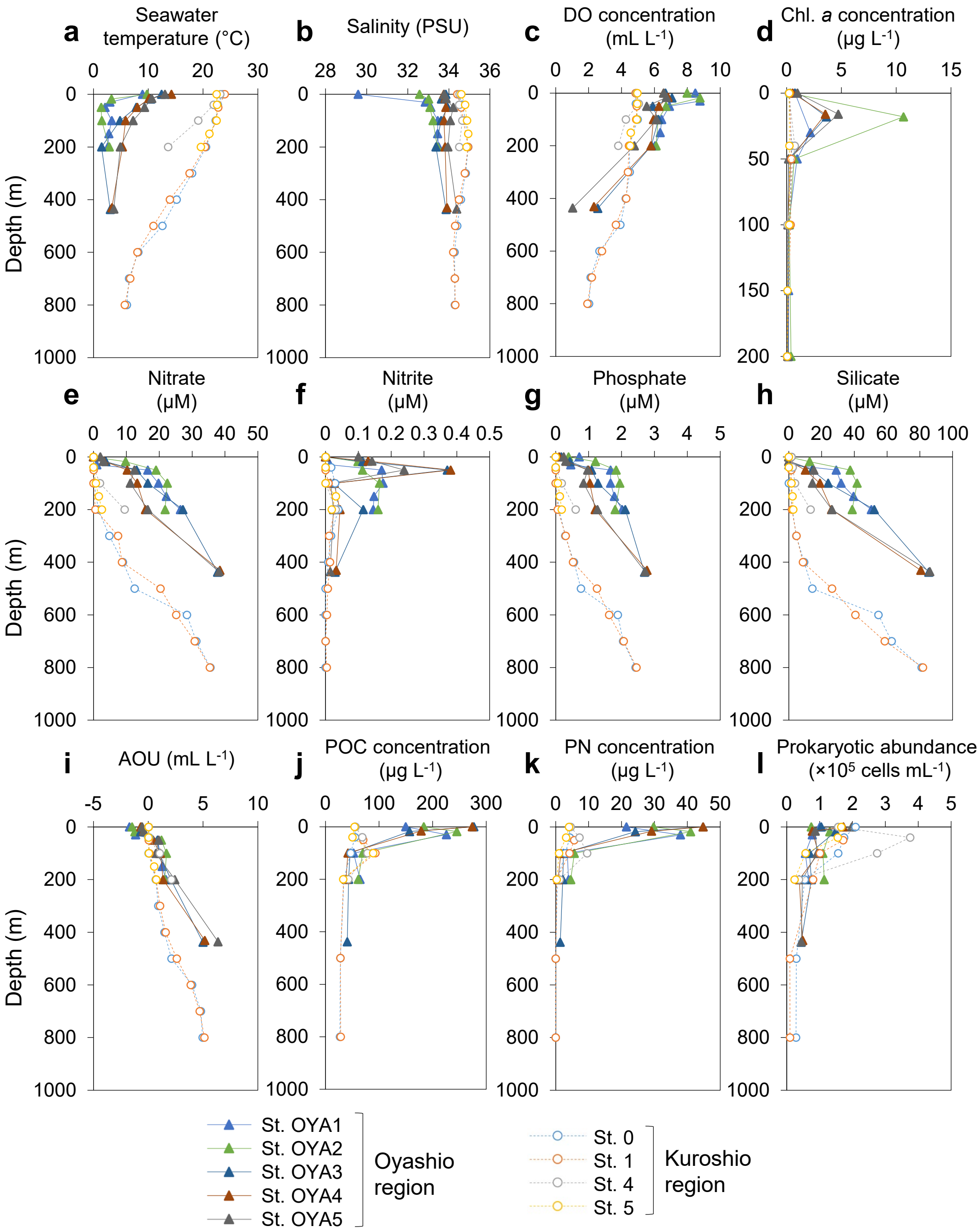

Figure S1.
Vertical profiles of environmental factors in the Oyashio and Kuroshio regions. The data of Kuroshio region were referred to Tada et al., 2020.

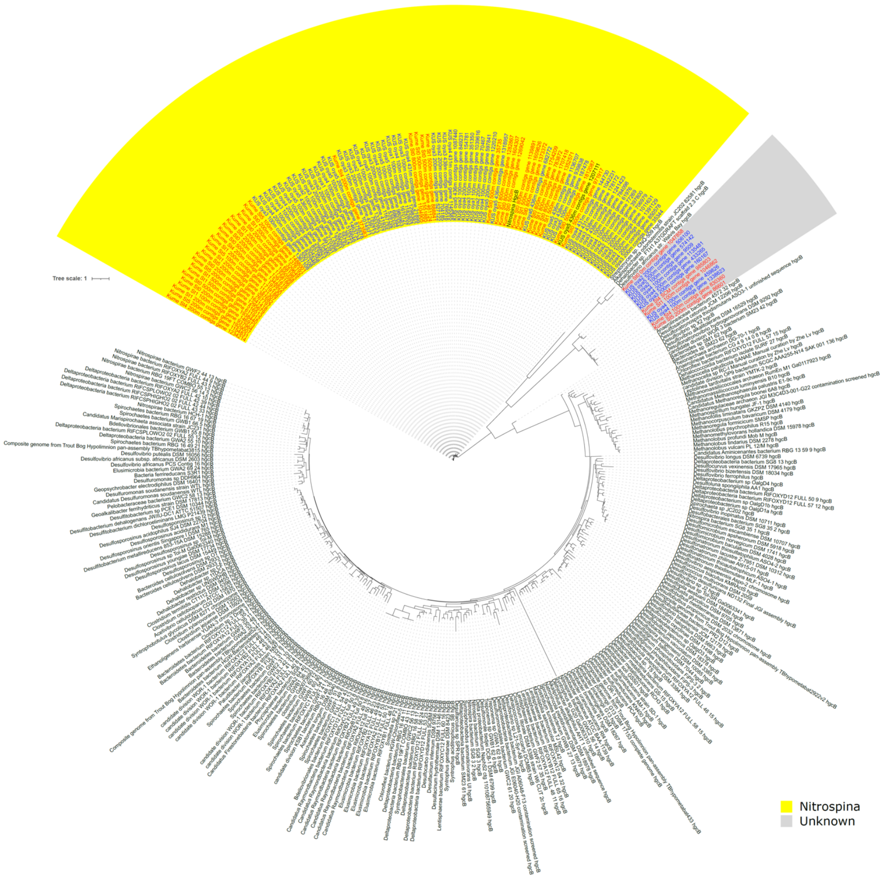

Figure S2.

Phylogenetic tree of *hgcB* genes detected in the Oyashio and Kuroshio regions.
Blue and red letters indicated the genes detected in the Oyashio and Kuroshio regions, respectively. The data of Kuroshio region were referred to Tada et al., 2020.

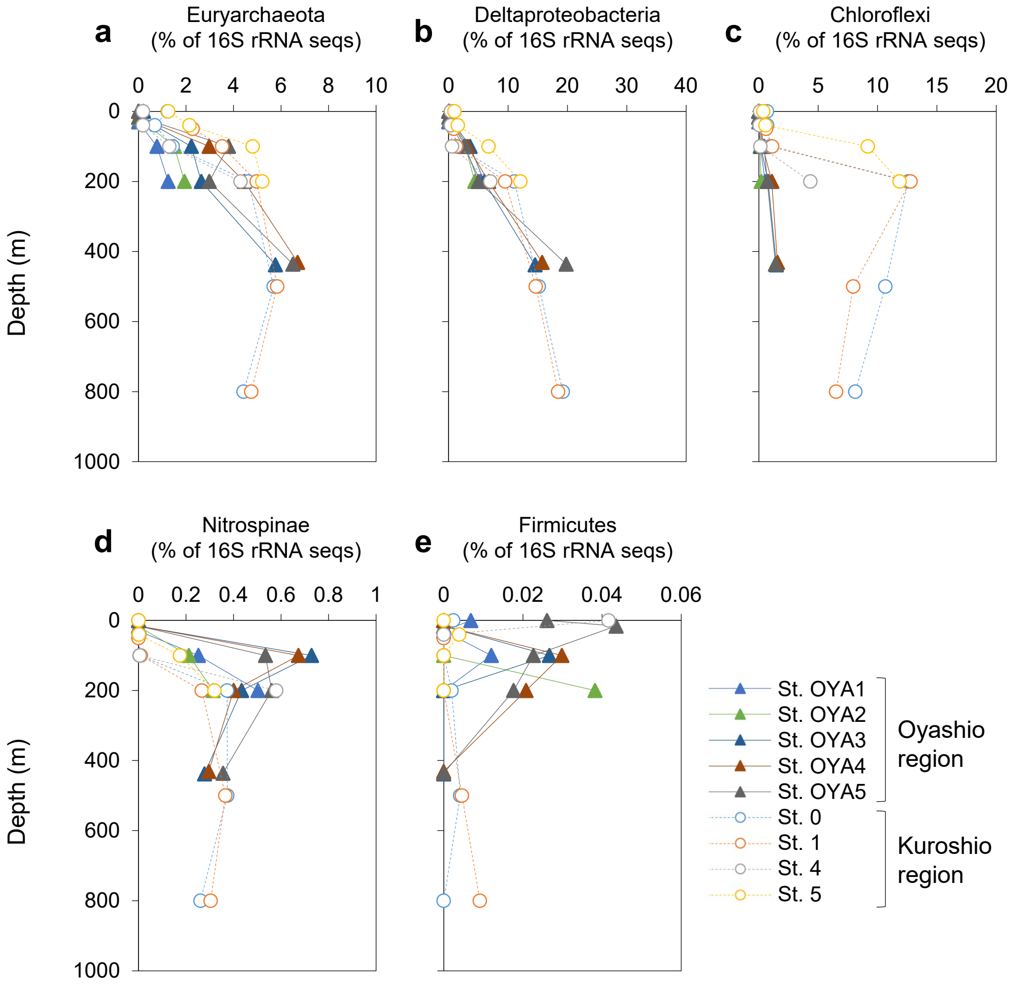

Figure S3.
Vertical distribution of major phylogenetic lineages involved in the confirmed and predicted mercury methylators including Euryarchaeota (a), Deltaproteobaccteria (b), Chloroflexi (c), Nitrospinae (d), and Firmicutes (e). The data of Kuroshio region were referred to Tada et al., 2020.

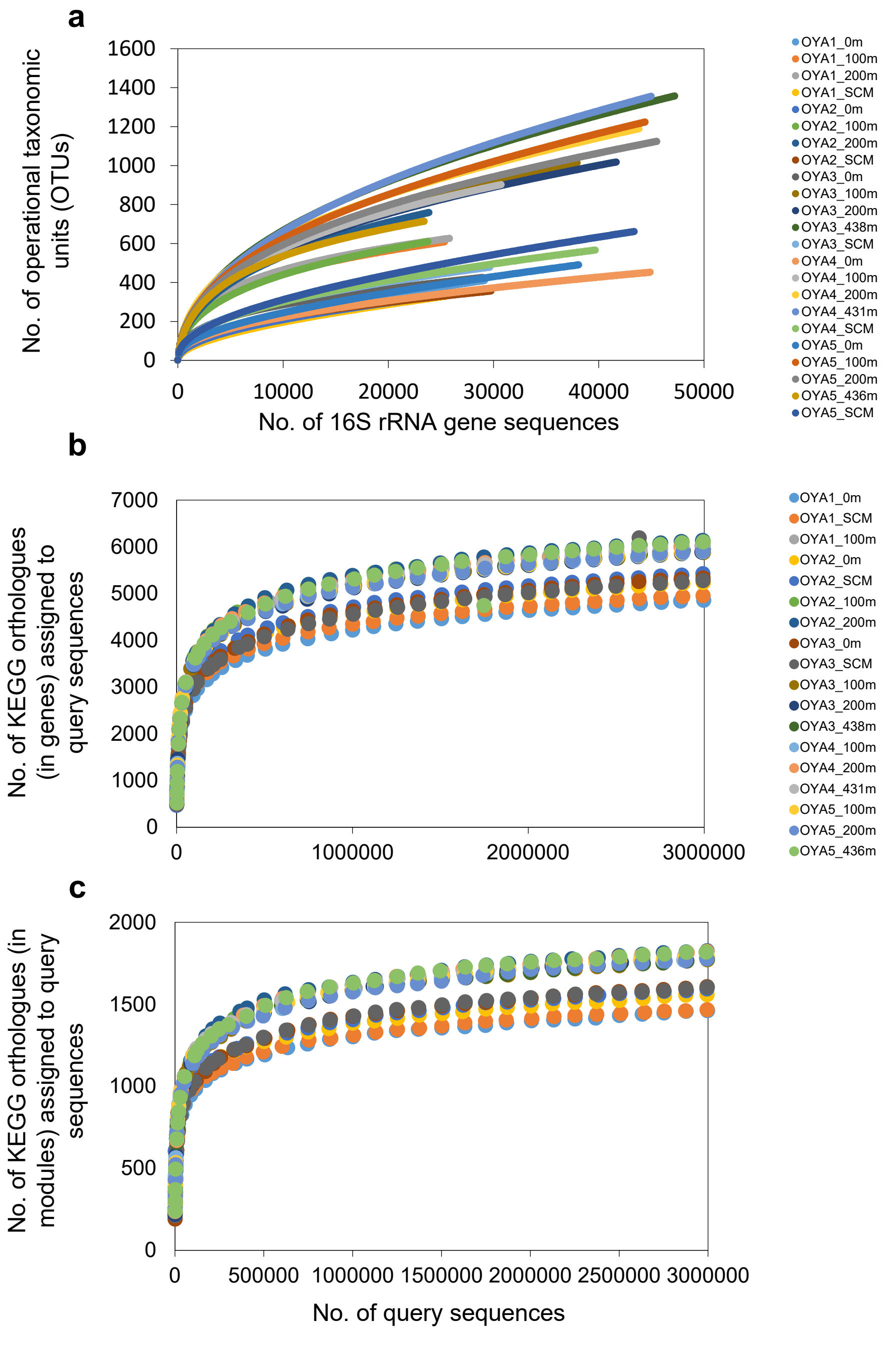

Figure S4.
Rarefaction curve for operational taxonomic units (OTUs) of 16S rRNA genes (a), the number of KEGG orthologues in genes (b), and modules (c) assigned to query sequences in the Oyashio region. The OTUs were classified by >97% similarity.

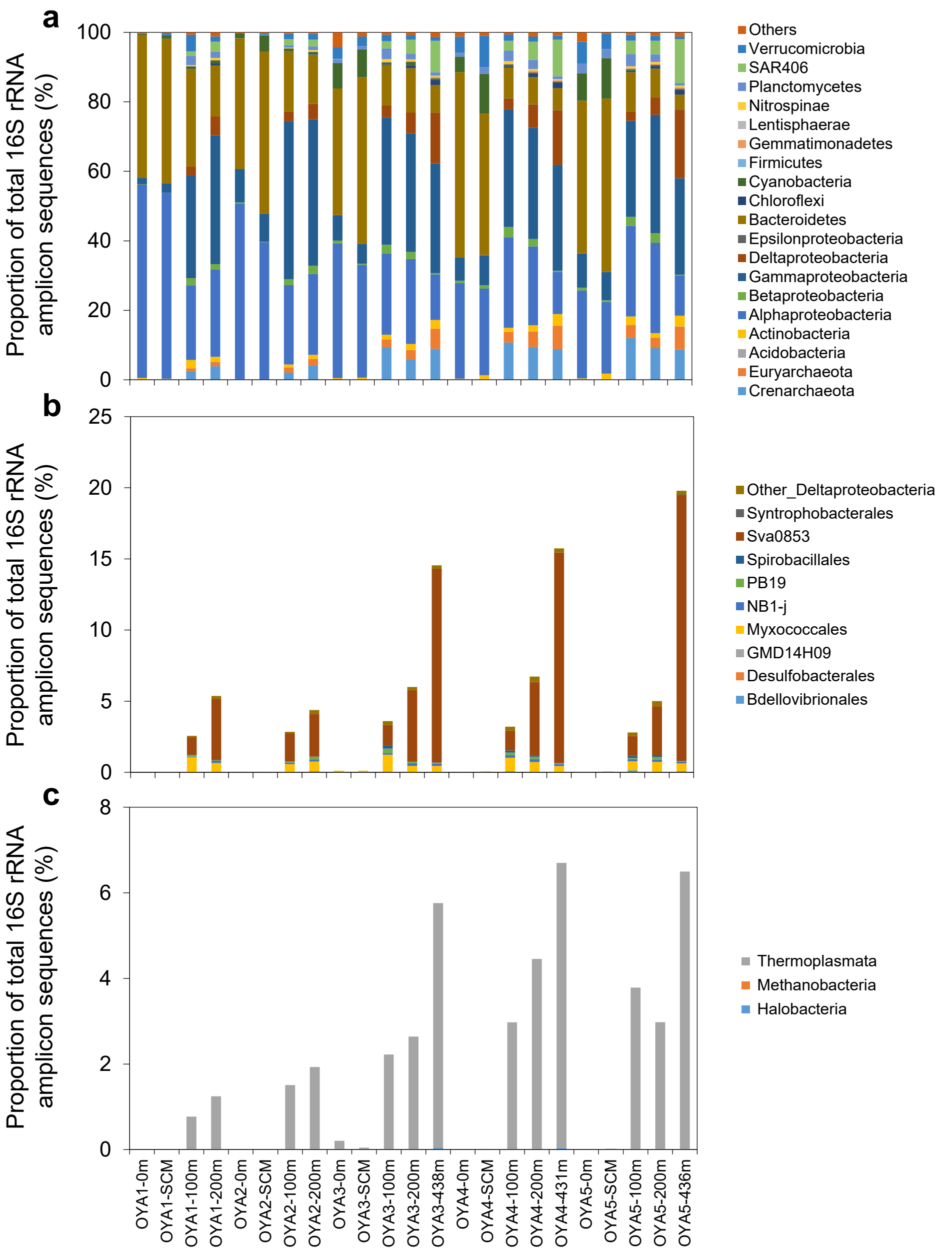

Figure S5.
The relative abundance of phylogenetic lineages (a), deltaproteobacterial (b) and euryarchaeal (c) clades to total 16S rRNA sequences in the Oyashio region.
