# Supplementary material for: Nitrospina-like Bacteria Are Dominant Potential Mercury Methylators in Both the Oyashio and Kuroshio Regions of the Western North Pacific
Source: Microbiol Spectr. 2021 Sep 8;9(2):e00833-21. doi: 10.1128/Spectrum.00833-21 (PMC8557936; doi:10.1128/Spectrum.00833-21)
Supplement: SUPPLEMENTAL FILE 3 — Supplemental material. Download SPECTRUM00833-21_Supp_3_seq6.pdf PDF file, 0.1 MB. [file spectrum00833-21_supp_3_seq6.pdf]

Supporting information

Seawater sampling and analytical procedures for environmental parameters

Seawater samples for macronutrients were collected in acrylic tubes (10 mL) and stored at -30°C until further analysis. Concentrations of nitrate plus nitrite (NO_3 + NO_2), phosphate (PO_4), and silicic acid [$Si(OH)_4$] were determined by using a QuAAtro39 analyzer (Bran + Luebbe). Particulate organic carbon (POC) and particulate nitrogen (PN) in the seawater were filtered onto pre-combusted (450°C for 4 h) GF/F filters (25 mm diameter, Whatman), and stored at -30°C until further analysis. These filters were treated with HCl fumes in a container for 24 h to remove inorganic carbon prior to the analyses (Hedges and Stern, 1984). The concentrations of POC and PN were measured by a FLASH EA1112 elemental analyzer (Thermo Finnigan). Physicochemical parameters of seawater, including water temperature, salinity, and chlorophyll *a* (Chl. *a*) and dissolved oxygen (DO) concentrations, were measured using a CTD system (RINKO-Profiler; JFE Advantec Co., Ltd). We determined a depth of the subsurface chlorophyll maximum (SCM) layer for each location on site based on the measured vertical profiles of the Chl. *a* concentrations. Apparent oxygen utilization (AOU) was calculated by the differences between DO and equilibrium oxygen concentrations.

DNA extraction

The collected seawater samples were passed through the Sterivex cartridge filters, followed by DNA extraction using an enzyme/phenol-chloroform protocol (Boström et al., 2004; Tada and Suzuki, 2016). Prokaryotic cells were lysed by lysozyme, proteinase K and SDS. The lysate was extracted twice with an equal volume of phenol-chloroform-isoamyl alcohol (25:24:1) and once with an equal volume of chloroform-isoamyl alcohol (24:1). DNA was precipitated with isopropanol and ammonium acetate. After treatment DNA solutions with RNase, the RNase were removed by the NucleoSpin gDNA clean-up kit (Takara, Co.). The extracted DNA was stored at -80°C until further analysis. The quality of the metagenomic DNA was assessed by 1% agarose gel electrophoresis.

References

Hedges JI and Stern JH. 1984. Carbon and nitrogen determinations of carbonate-containing solids. Limnol Oceanogr 29:657-663. doi: 10.4319/lo.1984.29.3.0657

Boström KH, Simu K, Hagström Å, Riemann L. 2004. Optimization of DNA extraction for quantitative marine bacterioplankton community analysis. Limnol Oceanogr-Meth 2:365–373.

Tada Y, Suzuki K. 2016. Changes in the community structure of free-living heterotrophic bacteria in the open tropical Pacific Ocean in response to microalgal lysate-derived dissolved organic matter. FEMS Microbiol Ecol 92:fiw099.

Tada Y, Marumoto K, Takeuchi A. 2020. Nitrospina-like bacteria are potential mercury methylators in the mesopelagic zone in the East China Sea. Front Microbiol 11: 1369.
